# Sex Differences Between Female and Male Newborn Piglets During Asphyxia, Resuscitation, and Recovery

**DOI:** 10.3389/fped.2019.00290

**Published:** 2019-07-16

**Authors:** Ramin P. La Garde, Po-Yin Cheung, Maryna Yaskina, Tze-Fun Lee, Megan O'Reilly, Georg M. Schmölzer

**Affiliations:** ^1^Medical University of Vienna, Vienna, Austria; ^2^Neonatal Research Unit, Centre for the Studies of Asphyxia and Resuscitation, Royal Alexandra Hospital, Edmonton, AB, Canada; ^3^Department of Pediatrics, University of Alberta, Edmonton, AB, Canada

**Keywords:** infants, newborn, neonatal resuscitation, chest compressions, asphyxia, sex differences

## Abstract

**Background:** Male and female newborns have differences in their fetal development, fetal-to-neonatal transition, and postnatal morbidity. However, the cardiovascular fetal-to-neonatal adaption is similar between sexes. No study has examined sex differences in newborns during hypoxia, asphyxia, cardio-pulmonary resuscitation, or post-resuscitation recovery.

**Methods:** Secondary analysis (two previous publications and two studies currently under peer-review) of 110 term newborn mixed breed piglets (1–3 days of age, weighing 2.0 ± 0.2 kg), which were exposed to 30 min normocapnic hypoxia followed by asphyxia until asystole, which was achieved by disconnecting the ventilator and clamping the endotracheal tube. This was followed by cardio-pulmonary resuscitation. For the analysis piglets were divided into female and male groups. Cardiac function, carotid blood flow, and cerebral and renal oxygenation were continuously recorded throughout the experiment.

**Results:** A total of 35/41 (85%) female and 54/69 (78%) male piglets resuscitated achieved ROSC (*p* = 0.881). The median (IQR) time to achieve return of spontaneous circulation in females and males was 111 (80–228) s and 106 (80–206) s (*p* = 0.875), respectively. The 4-h survival rate was similar between females and males with 28/35 (80%) and 49/54 (91%) piglets surviving (*p* = 0.241), respectively.

**Conclusions:** No difference between female and male newborn piglets was observed during hypoxia, asphyxia, resuscitation, and post-resuscitation recovery.

## Introduction

Male and female newborns have differences in their fetal development, fetal-to-neonatal transition, and postnatal morbidity (1–16). Male infants are more commonly born premature (12), have neonatal respiratory disease (e.g., respiratory distress or chronic lung disease) (5), higher newborn and infant mortality (4, 12), and worse long-term outcomes (13). The mortality gap in male and female newborns is decreasing since the 1970s, which is credited to better obstetric and neonatal care (17). However, the factors causing sex disparity in newborn infants remain unknown. Indeed, female infants are born with improved organ maturation, which translates into improved lung development (18), surfactant phospholipid composition, and surfactant function (2, 3, 13). As a result, male preterm lambs have a twice as high mortality during the first 8 h after birth compared to female preterm lambs (2, 3)—a result of cardiorespiratory failure. Interestingly, the male disadvantage cannot be explained by difference in the cardiovascular fetal-to-neonatal adaption (1).

Among survivors, birth asphyxia is the leading cause of neonatal neurologic morbidity, mortality, and long-term neurodevelopmental dysfunctions. The Nationwide Inpatient Sample Database, which includes infants >36 weeks' gestation and >2,500 g from over 1,000 hospitals in the United States (19), reported a male to female ratio for mild or moderate asphyxia (0.97 vs. 0.83%) and severe asphyxia (0.17 vs. 0.15%), respectively (19). Despite this male to female ratio, mortality was not associated to sex. In comparison, adult women have a higher rate of survival until hospital admission, better 1 month survival, and neurological outcome compared to men after out-of-hospital cardiac arrest (20, 21). Until now, sex differences in newborn infants during hypoxia, asphyxia, cardio-pulmonary resuscitation, or post-resuscitation recovery remains unknown. The aim was to examine sex differences between female and male newborn piglets during hypoxia, asphyxia, cardio-pulmonary resuscitation (CPR), or post-resuscitation recovery. We hypothesized that female newborn piglets have a faster return of spontaneous circulation (ROSC) and lower mortality compared to male newborn piglets.

## Methods

Secondary analysis of two previous publications (22, 23) and two studies currently under review examining 18, 21, and 100% oxygen during chest compression. A total of 110 term newborn mixed breed piglets (1–3 days of age, weighing 2.0 ± 0.2 kg) were obtained on the day of experimentation from the University Swine Research Technology Center. All experiments were conducted in accordance with the guidelines and approval of the Animal Care and Use Committee (Health Sciences), University of Alberta, presented according to the ARRIVE guidelines (24), and the study was registered at the preclinical trial registry PCTE 000139 (https://www.preclinicaltrials.eu).

### Animal Preparation

Piglets were instrumented as previously described with modifications (25–27). Following the induction of anesthesia using isoflurane, piglets were intubated via a tracheostomy, and pressure-controlled ventilation (Acutronic Fabian HFO; Hirzel, Switzerland) was commenced at a respiratory rate of 16–20 breaths/min and pressure of 20/5 cmH_2_O. Oxygen saturation was kept within 90–100%, glucose level and hydration was maintained with an intravenous infusion of 5% dextrose at 10 mL/kg/h. During the experiment anesthesia was maintained with intravenous propofol 5–10 mg/kg/h and morphine 0.1 mg/kg/h. Additional doses of propofol (1–2 mg/kg) and morphine (0.05–0.1 mg/kg) were also given as needed. The piglet's body temperature was maintained at 38.5–39.5°C using an overhead warmer and a heating pad.

### Hemodynamic Parameters

A 5-French Argyle^®^ (Klein-Baker Medical Inc. San Antonio, TX) double-lumen catheter was inserted via the right femoral vein for administration of fluids and medications. A 5-French Argyle^®^ single-lumen catheter was inserted above the right renal artery via the femoral artery for continuous arterial blood pressure monitoring in addition to arterial blood gas measurements. The right common carotid artery was also exposed and encircled with a real-time ultrasonic flow probe (2 mm; Transonic Systems Inc., Ithica, NY) to measure cerebral blood flow.

Piglets were placed in supine position and allowed to recover from surgical instrumentation until baseline hemodynamic measures were stable (minimum of 1 h). Ventilator rate was adjusted to keep the partial arterial CO_2_ between 35 and 45 mmHg as determined by periodic arterial blood gas analysis. Mean systemic arterial pressure, systemic systolic arterial pressure, heart rate, and percutaneous oxygen saturation were continuously measured and recorded throughout the experiment with a Hewlett Packard 78833B monitor (Hewlett Packard Co., Palo Alto, CA).

### Experimental Protocol for Included Studies

Study 1: Piglets were randomized into four groups: 2:1, 3:1, 4:1 compression:ventilation (C:V) ratio study: Piglets were randomized into four groups: 2:1 C:V group, 3:1 C:V group, 4:1 C:V group, or Sham (22). Study 2: Piglets were randomized into four groups: Chest compression during sustained inflation (CC+SI) with an duration of the sustained inflation of 20 s (CC+SI 20), or sustained inflation duration of 60 s (CC+SI 60), 3:1 C:V group, or Sham (23). Study 3: Piglets were randomized into four groups: 3:1 C:V with either with 18% oxygen, 21% oxygen, or 100% oxygen, or Sham. Study 4: Piglets were randomized into four groups: CC+SI+18%O_2_, CC+SI+21%O_2_, CC+SI+100%O_2_, or sham.

#### For All Studies

To reduce the occurrence of selection bias, a two-step randomization process was used. Following surgical instrumentation and the stabilization procedure, a subsequently numbered, sealed opaque envelope containing the assignment “sham” or “intervention was opened (step one). Piglets that were randomized to “intervention” underwent both hypoxia and asphyxia, whereas, the piglets randomized to “sham” were not. Sham-operated groups received the same surgical protocol, stabilization, and equivalent experimental periods without hypoxia and asphyxia. Upon meeting the criteria for CPR, a second sequentially numbered, sealed opaque envelope, containing the assignment for each study group (step two). The piglets that were randomized to “intervention” were exposed to 30–50 min of normocapnic hypoxia. The piglet was then disconnected from the ventilator and the endotracheal tube was clamped until total cardiac arrest. Ten seconds after cardiac arrest, positive pressure ventilation (PPV) was commenced for 30 s with a Neopuff T-Piece (Fisher & Paykel, Auckland, New Zealand). The default settings were a peak inflating pressure of 30 cmH_2_O, a positive end expiratory pressure of 5 cmH_2_O, and a gas flow of 8 L/min. CC were performed using the two-thumb encircling technique by a single operator (GMS) in all piglets. A metronome was used to achieve the targeted CC rate. After 30 s of CC, 100% oxygen was commenced for Study 1 & 2). In study 3 & 4 piglets were assigned to either “18% oxygen,” “21% oxygen,” or “100% oxygen” throughout the resuscitation. Epinephrine (0.01 mg/kg per dose) was administered intravenously 2 min after the start of PPV, and administered every 3-min as needed if no ROSC was observed. Epinephrine was administered to a maximum of four doses. ROSC was defined as an unassisted heart rate >100 bpm, which was accessed by ECG, for 15 s. After ROSC, piglets were allowed to recover for 4 h before the piglets were euthanized with an intravenous overdose of phenobarbital (100 mg/kg).

### Data Collection and Analysis

Demographics of study piglets were recorded. Transonic flow probes, heart rate and pressure transducer outputs were digitized and recorded with LabChart^®^ programming software (ADInstruments, Houston, TX). Post-mortem, lung tissue was cut from the right basal part of the lung lobe and clamp frozen in liquid nitrogen before storing at −80°C; the brain was removed from the skull and placed in ice-cold 2-methylbutane for 10 min before storing at −80°C. However, only lung and brain tissue samples from surviving piglets 4 h after intervention were collected at post-mortem (sham female *n* = 7, sham male *n* = 23, intervention female = 28, intervention male *n* = 49). Lung and brain tissue samples were homogenized in a lysis buffer (0.5% Tween-20/PBS containing a protease inhibitor cocktail). Homogenized samples were centrifuged at 3,000 × g for 10 min at 4°C. The supernatants were retained, and protein concentration was quantified using the Bradford method. Evidence of lung and brain injury was determined by quantification of the concentrations of the pro-inflammatory cytokines interleukin (IL)-1β,−6,−8, and tumor necrosis factor (TNF)-α in lung tissue homogenates using commercially available ELISA kits (PLB00B, P6000B, P8000, PTA00, R&D Systems, Minneapolis, USA). Cytokine concentrations were quantified according to protocols provided by the manufacturer and were expressed relative to protein concentration.

### Statistical Analysis

Data are presented as mean ±standard deviation (±SD) for normally distributed continuous variables and median (interquartile range—IQR) when the distribution was skewed. For all respiratory parameters, continuous values during CPR were analyzed. The data was tested for normality and compared using Student's *t-test* for parametric and Mann-Whitney *U*-test for non-parametric comparisons of continuous variables, and χ^2^ for categorical variables. To account for the effect of clustering (data coming from different studies) linear mixed models with random effects were used to examine the effect of sex on continuous outcome variables (time to ROSC). Bivariable generalized linear mixed models with binary outcome were used to ascertain the effect of sex on binary outcome variable (achieving ROSC, survival at 4 h after ROSC). Models were adjusted for age and weight to examine the combine effect on the outcome. *P*-values are 2-sided and *p* < 0.05 was considered statistically significant. Statistical analyses were performed with SigmaPlot (Systat Software Inc, San Jose, USA) and SAS Ver. 9.4 (SAS Institute Inc., Cary, NC, USA).

## Results

One hundred ten newborn mixed breed piglets were obtained on the day of the experiment and were subjected to hypoxic and asphyxiating protocol of the specific study. Baseline parameters were similar between sexes (Tables 1, 2) and arterial blood gas samples (pH, PaO_2_, PaCO_2_, base excess, HCO_3_, and lactate) at baseline, after asphyxiation, and at 4-h between female and male piglets (Table 3).

**Table 1:** Study characteristics of the four included studies.

**Study**	**Study-group**	**Study-group**	**Study-group**	**Primary outcome**
Different C:V ratios	2:1 C:V	3:1 C:V	4:1 C:V	Time to ROSC
Length of SI during CC+SI	3:1 C:V	CC+SI +20sec SI	CC+SI +60sec SI	Time to ROSC
Different oxygen concentration during 3:1 C:V ratio	3:1 C:V +18% Oxygen	3:1 C:V +21% oxygen	3:1 C:V +100% oxygen	Time to ROSC
Different oxygen concentration during CC+SI	CC+SI + 18% O_2_	CC+SI +21% oxygen	CC+SI +100% oxygen	Time to ROSC

*C:V, Compression:Ventilation; SI, Sustained Inflation; CC+SI, Chest Compression during Sustained inflation; ROSC, Return of Spontaneous Circulation*.

**Table 2:** Characteristics of asphyxia, resuscitation and survival of asphyxiated piglets.

		**Female (*n* = 41)**	**Male (*n* = 69)**	***p*-value**
Asphyxia time (sec)^†^		292 (221–424)	329 (205–501)	0.436
Immediately before resuscitation	pH^†^	6.6 (6.5–6.7)	6.6 (6.5–6.7)	0.884
	paCO_2_ (mm Hg)^†^	97 (80–115)	101 (79–115)	0.956
	Lactate (mmol/L)^†^	15.5 (14.2–17.7)	15.7 (14.4–16.9)	0.926
	Base excess (mEq/L)	−27 (−29–24)	−26 (−28–24)	0.360
Resuscitation	Received 100% oxygen	25 (61)	40 (58)	0.996
	Epinephrine doses (n)^†^	1.0 (0–2)	1.0 (0–3)	0.704
Achieving ROSC		35 (85)	54 (78)	0.881
ROSC time (sec)^†^		111 (80–228)	106 (80–206)	0.875
Survival 4h after ROSC		28/35 (80)	49/54 (91)	0.241

Data are presented as n (%), unless indicated

† *median (IQR)*.

**Table 3:** Blood gas changes before and after resuscitation.

	**Female (*n* = 41)**	**Male (*n* = 69)**	***p*-value**
**pH**			
Baseline	7.48 (0.08)	7.48 (0.08)	0.923
After asphyxiation	6.65 (0.16)	6.64 (0.14)	0.643
4 h after reoxygenation	7.30 (0.14)	7.26 (0.14)	0.17
**PaO**_**2**_ **(mm Hg)**			
Baseline	83 (17)	85 (13)	0.506
After asphyxiation	12 (9)	11 (7)	0.556
4 h after reoxygenation	86 (22)	86 (24)	0.965
**PaCO**_**2**_ **(mm Hg)**			
Baseline	34 (7)	35 (5)	0.554
After asphyxiation	96 (25)	97 (22)	0.855
4 h after reoxygenation	39 (7)	41 (11)	0.293
**Base excess (mEq/L)**			
Baseline	2 (4)	2 (3)	0.497
After asphyxiation	−25 (5)	−26 (3)	0.889
4 h after reoxygenation	−7 (6)	−9 (7)	0.266
**HCO**_**3**_ **(mm Hg)**			
Baseline	26 (3)	25 (2)	0.439
After asphyxiation	11 (3)	11 (2)	0.905
4 h after reoxygenation	19 (5)	18 (5)	0.378
**Lactate (mmol/L)**			
Baseline	3.6 (0.9)	3.9 (1.2)	0.141
After asphyxiation	15.5 (2.6)	15.7 (1.9	0.702
4 h after reoxygenation	6.0 (3.5)	7.1 (3.9)	0.235

*Data are presented as mean (SD)*.

**Figure 1:**
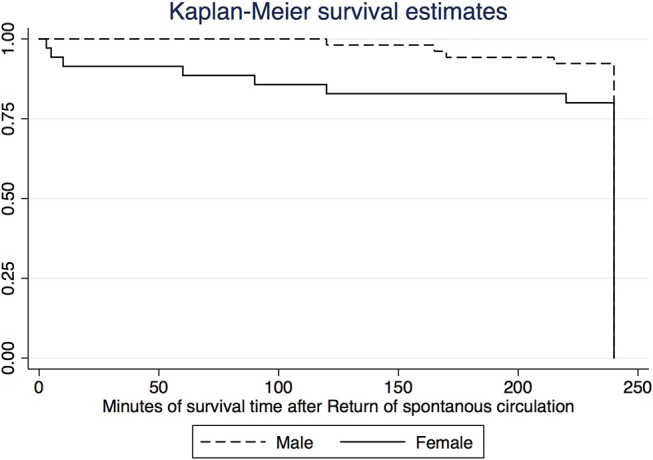
Kaplan-Meier survival curve.

Duration of asphyxia, the degree of asphyxiation (as indicated by pH, PaCO_2_, Base excess, and lactate) were similar between female and male piglets (Table 2). A total of 35/41 (85%) female and 54/69 (78%) male piglets achieved ROSC (Table 2). Median (IQR) time to achieve ROSC in females and males was 111 (80–228) s and 106 (80–206) s, respectively (Table 2). During CPR, 25/41 and 40/69 female and male piglets required 100% oxygen. The 4-h survival rate after ROSC was similar between females and males with 28/35 (80%) and 49/54 (91%) piglets surviving, respectively. Sex was not statistically significant in models with time to achieve ROSC, achieving ROSC, and survival 4 h after ROSC as outcome variables, both as a single independent variable and adjusted for age and weight. Results from models are presented in Table 4. A Kaplan-Meier survival curve is presented in Figure 1.

**Table 4:** Results from generalized linear mixed models (binary outcome) or linear mixed models (continuous outcome).

**Independent variable**	**Univariate models (independent variable: sex)**		**Sex adjusted for age and weight**	
	**OR (95% CI)**	***p*-value**	**OR (95% CI)**	***p*-value**
Achieving ROSC	1.62 (0.30, 8.80)	0.43	1.79 (0.31, 10.22)	0.37
Survival 4 h after ROSC	1.90 (0.36, 10.17)	0.31	2.08 (0.37, 11.66)	0.27
	**Estimate (95% CI)**	***p*****-value**	**Estimate (95% CI)**	***p*****-value**
1/(Time to achieve ROSC), 1/sec^*^	0.0001 (−0.003, 0.003)	0.93	0.0004 (−0.003, 0.003)	0.68

* *A transformation was used since residuals were not normally distributed*.

### Changes in Hemodynamic Parameters

**Figure 2:**
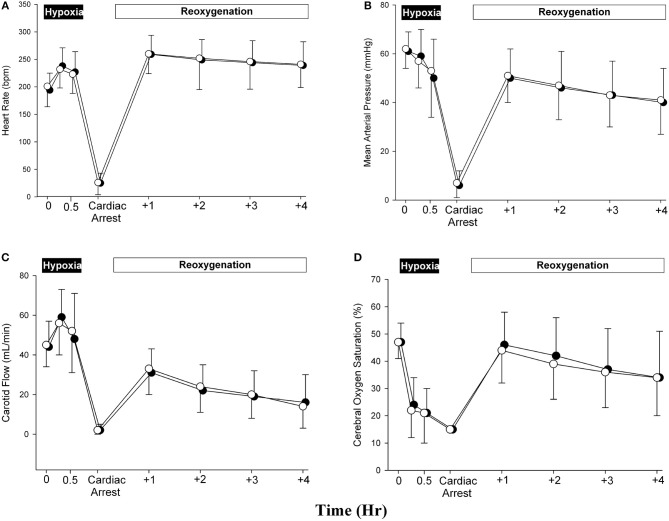
Changes in **(A)** heart rate [beats per minute (bpm)], **(B)** mean arterial blood pressure (mmHg), **(C)** carotid blood flow (mL/min), and **(D)** cerebral oxygen saturation (%) during hypoxia, asphyxia, and post-resuscitation (solid circle represents male and open circle represents female).

In both female and male piglets, heart rate increased during hypoxia and maintained steadily throughout the remaining experimental period (Figure 2A; Table 5). As shown in Figure 2B and Table 3, the mean blood pressure of both female and male piglets decreased over time and was similar between female and male piglets throughout the experimental period.

**Table 5:** Hemodynamic changes before and after resuscitation.

	**Female (*n* = 41)**	**Male (*n* = 69)**	***p*-value**
**HEART RATE (beats/min)**			
Baseline	201 (37)	194 (31)	0.254
After hypoxia	223 (35)	227 (37)	0.529
4 h after reoxygenation	241 (42)	239 (43)	0.858
MAP (mm Hg)			
Baseline	62 (8)	61 (8)	0.499
After hypoxia	53 (19)	50 (16)	0.369
4 h after reoxygenation	41 (14)	40 (14)	0.733
**CAROTID FLOW (mL/min)**			
Baseline	45 (10)	44 (13)	0.683
After hypoxia	52 (21)	48 (22)	0.368
4 h after reoxygenation	14 (11)	16 (13)	0.425
**CEREBRAL OXYGENATION (%)**			
Baseline	47 (6)	47 (7)	0.841
After hypoxia	21 (11)	20 (9)	0.968
4 h after reoxygenation	34 (14)	34 (17)	0.992
**RENAL OXYGENATION (%)**			
Baseline	51 (13)	54 (12)	0.215
After hypoxia	16 (3)	17 (6)	0.368
4 h after reoxygenation	45 (15)	39 (17)	0.102

*Data are presented as mean (SD)*.

### Changes in Cerebral Perfusion

The mean common carotid arterial flow of both female and male piglets the sham group decreased with time and maintained about the same during the experimentation period (Figure 2C; Table 3). The crSO_2_ of both female and male piglets decreased from the baseline during hypoxia and then returned to baseline after ROSC, with a continuous decline over the 4-h recovery period (Figure 2D; Table 5).

### Injury Markers

**Figure 3:**
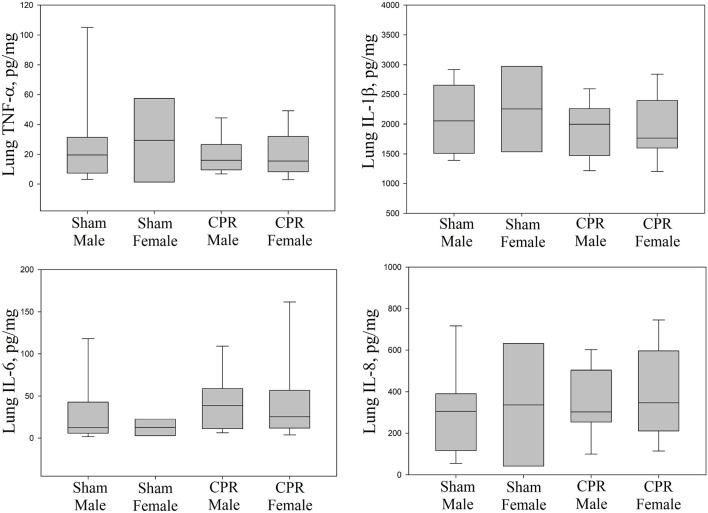
Proinflammatory cytokines in lung tissue. Concentrations of TNF-α, interleukin (IL)-1β, IL-6, and IL-8 in lung tissue homogenates, expressed relative to lung protein concentration, in sham female (*n* = 5) and male piglets (*n* = 13) and female (*n* = 28) and male (*n* = 49) piglets receiving resuscitation. Results represent the median (solid bar), IQR (box margin), and 95% confidence interval.

**Figure 4:**
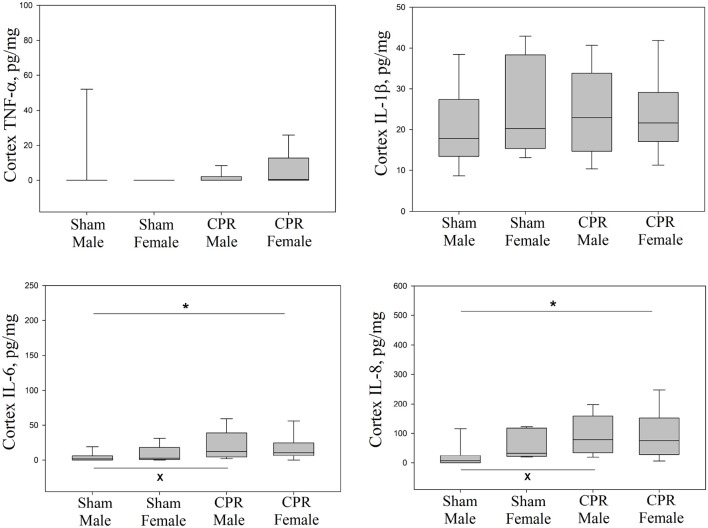
Concentrations of IL-1β, IL-6, and IL-8 in frontoparietal cortex homogenates, expressed relative to protein concentration, in sham female (*n* = 5) and male piglets (*n* = 13) and female (*n* = 28) and male (*n* = 49) piglets receiving resuscitation. Results represent the median (solid bar), IQR (box margin), and 95% confidence interval. Asterisk (*) indicates a significant difference between groups (*p* < 0.05).

**Figure 5:**
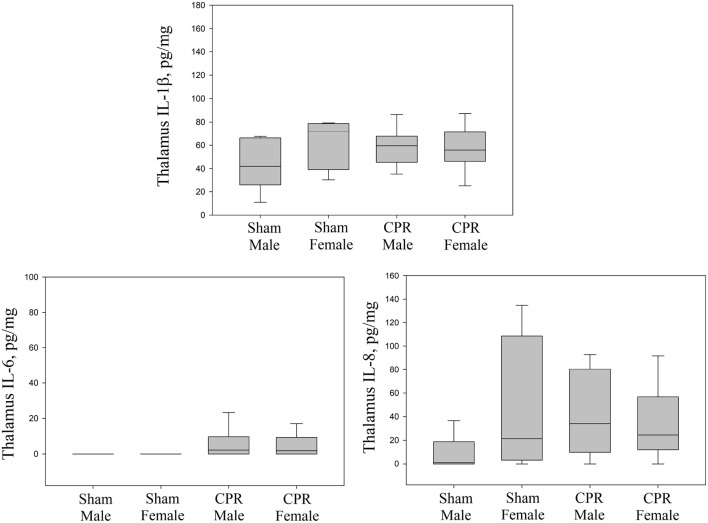
Concentrations of IL-1β, IL-6, and IL-8 in thalamus homogenates, expressed relative to protein concentration, in sham female (*n* = 5) and male piglets (*n* = 13) and female (*n* = 28) and male (*n* = 49) piglets receiving resuscitation. Results represent the median (solid bar), IQR (box margin), and 95% confidence interval.

Lung injury markers of IL-1β, IL-6, IL-8, and TNF-α in lung tissue homogenates between sham and female and male intervention groups were similar (Figure 3). Although, IL-6 and IL-8 in the cortex were statistically higher in female and male piglets compared to male sham; the injury markers (IL-1β, IL-6, and IL-8) were similar between female and male piglets (Figure 4). TNF-α concentration was beyond detection in the thalamus. IL-1β, IL-6, and IL-8 were similar between female and male piglets in front oparietal cortex (Figure 4) or thalamus (Figure 5).

## Discussion

This is the first study to directly compare female and male piglets and the potential impact on outcomes during asphyxia, resuscitation, and post-resuscitation recovery. Our study can be summarized as followed (i) time to ROSC and survival rates were similar between sexes (Table 2), (ii) four-hour survival rate was similar between females and males (Table 2), however female piglets died more quickly compared to their male counterparts throughout the 4-h recovery period (Figure 1), (iii) no difference in epinephrine use (Table 2), (iv) no differences in any hemodynamic measurements between sexes (Table 5), and (v) no differences in brain injury markers (Figures 4, 5).

Based on animal and epidemiological data, prematurely born males have worse short-term neonatal morbidities as well as long-term neonatal outcomes (1–13). In addition, there is a male disadvantage in infants >36 weeks' gestation who develop any degree (mild, moderate or severe) birth asphyxia compared to female infants (0.97 vs. 0.83% and 0.17 vs. 0.15%, respectively) (19). Although, there is a higher risk for male term infants to have birth asphyxia, the mortality is similar between sexes (19). These observations are similar to our data where no difference in time to ROSC and survival in male and female piglets (Table 2). Conversely, adult women have higher rates of survival until hospital admission, 1 month survival, and neurological outcome compared to men after out-of-hospital cardiac arrest (20, 21). While we observed female piglets to die more quickly compared to their male counterparts throughout the 4-h recovery period, we cannot comment on outcomes beyond the 4-h window, as all animals were euthanized at that time point (Figure 1).

A recent review by Lorente-Pozo et al, reported sex related differences during fetal to neonatal transition including (i) hemodynamic circulatory transition, (ii) breathing attempts, (iii) incidence of asphyxia, (iv) incidence of hypoxic ischemic encephalopathy, and (v) drug responses (13). While in our asphyxia model piglets have undergone fetal to neonatal transition, which prevents examination of differences during hemodynamic circulatory transition. In ventilated, anesthetized preterm lambs no sex differences during cardiopulmonary transition at birth was observed (1). Thus, it is controversial if an impaired cardiovascular transition at birth contributes to “neonatal male disadvantage” (1). Furthermore, as all our piglets were exposed to hypoxia, asphyxia, and chest compression, we can also not comment on the incidence of asphyxia and hypoxic ischemic encephalopathy. However, a recent database reported a male to female ratio for mild, moderate, or severe asphyxia (19).

Furthermore, male rats develop behavioral/neurocognitive deficits more often compared to female rate after asphyxia (28–31). The pathways causing these behavioral/neurocognitive deficits include (i) pro-apoptotic signaling (28–31), and (ii) caspase-independent cell death (28–31), which are very different between male and female rats. The pathophysiology of perinatal brain damage (e.g., asphyxia, prematurity, and infection) is linked to hypoxia-reoxygenation (13, 28–31). Similarly, a recent meta-analysis reported higher rates of cognitive/behavioral deficits in male rats compared to matched females rats with hypoxic ischemic encephalopathy (32). Interestingly, our model did not identify any differences in brain injury between the sexes (Figure 2). Our observations might be explained by the different models used (33). Unfortunately, the rat models used occlusion of the left common carotid artery followed by hypoxia, which resulted in a stroke on top of hypoxia, which is never present during hypoxic-ischemic events around birth (28–31). This certainly explains the observed differences.

## Limitations

Our use of a piglet asphyxia model is a great strength of this translational study, as this model closely simulates delivery room events, with the gradual onset of severe asphyxia leading to bradycardia (22, 23). In addition, the large number of piglets (*n* = 110) is a further strength of this study. However, several limitations should be considered: Our asphyxia model uses piglets that have already undergone the fetal to neonatal transition, and piglets were sedated/anesthetized (22, 23). Furthermore, our model requires piglets to be intubated with a tightly sealed endotracheal tube to prevent any endotracheal tube leak; this may not occur in the delivery room as mask ventilation is frequently used (22, 23). Nevertheless, our findings are still clinically relevant as the distribution of cardiac output in the fetus and post transitional neonate during asphyxia episodes are qualitatively similar (33). Our resuscitation model is slightly different from the currently recommended resuscitation guidelines, as we administered epinephrine 90 s after CC were initiated (34–36). This may have influenced our results. Nevertheless, there was no significant difference in the amount of epinephrine doses between both groups.

## Conclusion

No difference between female and male newborn piglets was observed during hypoxia, asphyxia, resuscitation, and post-resuscitation recovery. Epidemiological studies of human newborn infants are needed to validate these findings.

## Ethics Statement

All experiments were conducted in accordance with the guidelines and approval of the Animal Care and Use Committee (Health Sciences), University of Alberta, presented according to the ARRIVE guidelines (21), and the study was registered at the preclinical trial registry PCTE 000139 (https://www.preclinicaltrials.eu).

## Author Contributions

GS, P-YC, MO, T-FL, and RL: conception and design, collection and assembly of data, analysis and interpretation of the data, drafting of the article, critical revision of the article for important intellectual content, and Final approval of the article. MY: analysis and interpretation of the data, drafting of the article, critical revision of the article for important intellectual content, and Final approval of the article.

### Conflict of Interest Statement

The authors declare that the research was conducted in the absence of any commercial or financial relationships that could be construed as a potential conflict of interest.

## Abbreviations

CC: Chest compression
CPR: Cardiopulmonary resuscitation
ROSC: Return of spontaneous circulation
MAP: Mean arterial blood pressure.
